# Financial feasibility of end-user designed rainwater harvesting and greywater reuse systems for high water use households

**DOI:** 10.1007/s11356-017-8710-5

**Published:** 2017-03-30

**Authors:** Edgar Ricardo Oviedo-Ocaña, Isabel Dominguez, Sarah Ward, Miryam Lizeth Rivera-Sanchez, Julian Mauricio Zaraza-Peña

**Affiliations:** 1 Grupo de Investigación en Recursos Hídricos y Saneamiento Ambiental (GPH), Escuela de Ingeniería Civil, Facultad de Ingenierías Físico-Mecánicas, Universidad Industrial de Santander, Carrera 27 Calle 9, Bucaramanga, Colombia; 2 Centre for Water Systems, College of Engineering, Mathematics and Physical Sciences, University of Exeter, Kay Building, North Park Road, Exeter, EX4 4QF UK

**Keywords:** Alternative water supply systems, Colombia, End-user, Financial feasibility, Greywater reuse, Rainwater harvesting

## Abstract

Water availability pressures, competing end-uses and sewers at capacity are all drivers for change in urban water management. Rainwater harvesting (RWH) and greywater reuse (GWR) systems constitute alternatives to reduce drinking water usage and in the case of RWH, reduce roof runoff entering sewers. Despite the increasing popularity of installations in commercial buildings, RWH and GWR technologies at a household scale have proved less popular, across a range of global contexts. For systems designed from the top-down, this is often due to the lack of a favourable cost-benefit (where subsidies are unavailable), though few studies have focused on performing full capital and operational financial assessments, particularly in high water consumption households. Using a bottom-up design approach, based on a questionnaire survey with 35 households in a residential complex in Bucaramanga, Colombia, this article considers the initial financial feasibility of three RWH and GWR system configurations proposed for high water using households (equivalent to >203 L per capita per day). A full capital and operational financial assessment was performed at a more detailed level for the most viable design using historic rainfall data. For the selected configuration (‘Alt 2’), the estimated potable water saving was 44% (equivalent to 131 m^3^/year) with a rate of return on investment of 6.5% and an estimated payback period of 23 years. As an initial end-user-driven design exercise, these results are promising and constitute a starting point for facilitating such approaches to urban water management at the household scale.

## Introduction

Increased pressure over water resources to meet the demands of growing populations is pushing supply systems to their limits (Couto et al. 2015). Aspects such as the reduction of water availability from surface and groundwater sources, continued population growth (e.g. an increase of 1.8 million people between 2005 and 2030 is projected) (Muthukumaran et al. 2011) and climate variability, which has increased episodes of drought, contribute to intensify concerns about water availability. These threats and trends make urgent the need to adapt water management and governance to current and changing social and environmental conditions (Domènech and Saurí 2011; Tian et al. 2012). In this context, water conservation and efficiency gain significance, involving both the controlled and efficient use of water resources and measures for wastewater reuse (Couto et al. 2015).

Residential water use represents the sector with lower water consumption worldwide (12%), compared to agriculture (69%) and industry (19%) (FAO 2014). However, this sector is perhaps more responsive to the introduction of changes in water management that contribute to enhance the water efficiency of water-using everyday practices but not if oversimplified models of consumer behaviour are used (Hoolohan and Browne 2016). Water use in the residential sector depends, amongst other aspects, on cultural customs, economic development and water availability. However, general uses with higher consumption in developed or developing countries (i.e. Colombia, Brazil, Peru, USA and Germany) are toilet flushing, clothes washing, hand washing, personal hygiene (showers), food preparation and cleaning utensils (kitchen) and to a lesser extent house cleaning, own consumption (actual drinking) and garden irrigation (Cohim et al. 2009; Li et al. 2009; Mayer et al. 1999; Mourad et al. 2011; Seifert 2009; Silva et al. 2015; Suárez et al. 2006). This indicates that a high proportion of residential water uses do not require strictly drinking water quality—it is possible to use water with a lower quality for such uses. In Colombia, the amount of water for residential use that does not require drinking water quality could be approximately 71% (Suárez et al. 2006), being higher in households with high socioeconomic conditions (i.e. called strata 5 and 6), which are also characterised by higher water use levels (CRA 2001). At the opposite end of the demand spectrum, Sanches Fernandes et al. (2015) investigated rainwater harvesting (RWH) systems for low-demand applications in the Portuguese context. Through examination of water-saving efficiency and tank sizing using the Ripple method, they identified the impact of drought events on tank sizing for two irrigation scenarios. Fifty-percent reductions in tank size were viable, resulting in positive impacts on cost and return periods without compromising efficiency or increasing probability of failure to supply.

Various options have been proposed to reduce water use in the residential sector including the development of low water consumption technology (Vieira et al. 2015); awareness programs aimed at water use efficiency; and implementation of policies, such as higher-cost tariffs (Sahin et al. 2015). One alternative increasingly considered is water reuse through decentralised systems (Matos et al. 2014). This alternative involves capturing water (e.g. greywater or rainwater) from generation sites (such as showers or basins or from roofs) and then treating and distributing it for non-potable water uses in the household (e.g. general washing and gardening purposes or toilet flushing) (Kujawa and Zeeman 2006; Lee et al. 2016). This approach mainly considers RWH and greywater reuse (GWR), which have the greatest potential as complementary alternative water sources or supply systems (AWSSs) due to their reduced pollution compared to other alternatives (Stec and Kordana 2015; WHO-ROEM 2006).

Technical and financial feasibility assessments are a key strategy to promote and make viable GWR and RWH at the household level. However, the majority of past studies have focused on public buildings (Neto et al. 2012), office buildings (Ward et al. 2012; Motawa and Carter 2013), universities (Roebuck and Ashley 2007), residential complexes (Gardels 2011) and communal systems for RWH (Gurung and Sharma 2014). Few studies have addressed individual households, such as those reported by Ghisi and Oliveira (2007) in Brazil and Domènech and Saurí (2011) and Morales-Pinzón et al. (2014) in Spain. The most recent study identified, by Melville-Shreeve et al. (2016), focused on multiple configurations of RWH systems for the household scale. Although the study used a multicriteria analysis, it only included capital RWH system costs (i.e. it did not include network, treatment or operation and maintenance costs or broader financial indicators) and did not consider GWR. In developing countries such as Colombia, limited information on the financial assessment of individual decentralised systems was identified.

In this research, the financial feasibility of implementing a system that allows for RWH and GWR to be used in strictly non-drinking water residential uses is evaluated, considering social, technical and building-related conditions linked to acceptability. The research is situated in a typical high water using household in Bucaramanga (Colombia). This paper evaluates indicators such as the internal rate of return (IRR), net present value (NPV) and payback period (PP), comparing the proposed system and the conventional alternative (i.e. without GWR and RWH).

## Materials and methods

This section firstly outlines the study area in which the analysis is situated. Secondly, it describes the criteria used to design three AWSSs, including a questionnaire with residents in the study area. Thirdly, it identifies and explains the initial system screening criteria utilised to compare the three different systems, followed by detailed description of the selected AWSS focused on in subsequent analyses. Finally, it outlines the cost and financial criteria used to assess the financial feasibility of the selected AWSS.

### Study area

The study system was situated in an urban household located in the metropolitan area of Bucaramanga (Colombia) and classified as socioeconomic stratum 6 (strata 1 and 6 represent the lowest and highest socioeconomic levels, respectively). The household had three floors, a roof comprising Spanish clay tiles (101 m^2^), a patio (18 m^2^), a garden (21 m^2^) and five bathrooms, across a total built area of 216 m^2^. The house was equipped with a hydro-pneumatic pump system located on the third floor (next to a previously used drinking water storage tank) to ensure the pressure for the bathroom located on this floor. The average rainfall in the study area was 1053 mm/year and the temperature was 25°C (IDEAM 2015).

### Criteria for alternative RWH and GWR system designs

In addition to standard design criteria, such as the sizing of the tank, features affecting end-user acceptability and water quality should also be considered at the design stage. In this study, the focus was on social acceptability in relation to source water and willingness to undertake maintenance activity and water quality with regard to consideration of including a treatment system.

#### User acceptability

A questionnaire was developed to determine the types of criteria that may be important to householders in the design of a RWH or GWR system. The questionnaire had 14 questions that explored issues such as (i) average per capita water consumption, (ii) the device users would be willing to connect to an AWSS, (iii) participants’ willingness to undertake operation and maintenance activities of an eventual AWSS and (iv) the range of additional investment that users would be willing to pay for such a system.

Using the questionnaire, a survey was conducted in a residential complex of 115 households, belonging to socioeconomic stratum 6 and located in the metropolitan area of Bucaramanga. A random selection of households was not feasible because 30% of the households were empty at the time of the survey. In addition, in a further 40%, the householders were not willing to participate in the survey. Therefore, 100% of the households available and willing to participate at the time of the fieldwork were surveyed, representing 30% of the households in the residential complex (i.e. 35 households), which is an acceptable cooperation rate as recommended by Robson (2002).

From the water service bills provided by the residents who took part in the household survey (35 households), an average water consumption of 203 L per capita per day (lpcd) (±84 lpcd) was estimated. This value was higher than the average value reported for the country, which for high socioeconomic strata (i.e. 5 and 6) is 170 lpcd (CRA 2001). However, the case study is located in a region with warm temperatures (25°), which in addition to the high socioeconomic stratum could contribute to the observed water consumption levels being higher than the country average for the correspondent socioeconomic strata.

It was identified that 97 and 86% of participants were willing to use AWSS, such as rainwater and greywater, respectively. These results were similar to those obtained for GWR in Brazil (De Araujo Batista et al. 2015) and Oman (Prathapar et al. 2005), where 83 and 84%, respectively, expressed this willingness. However, there was greater acceptance for RWH compared with GWR due to hygiene concerns when greywater is used (100% of participants), as found by De Araujo Batista et al. (2015) in the city of Campinhas (Brazil).

**Fig. 1:**
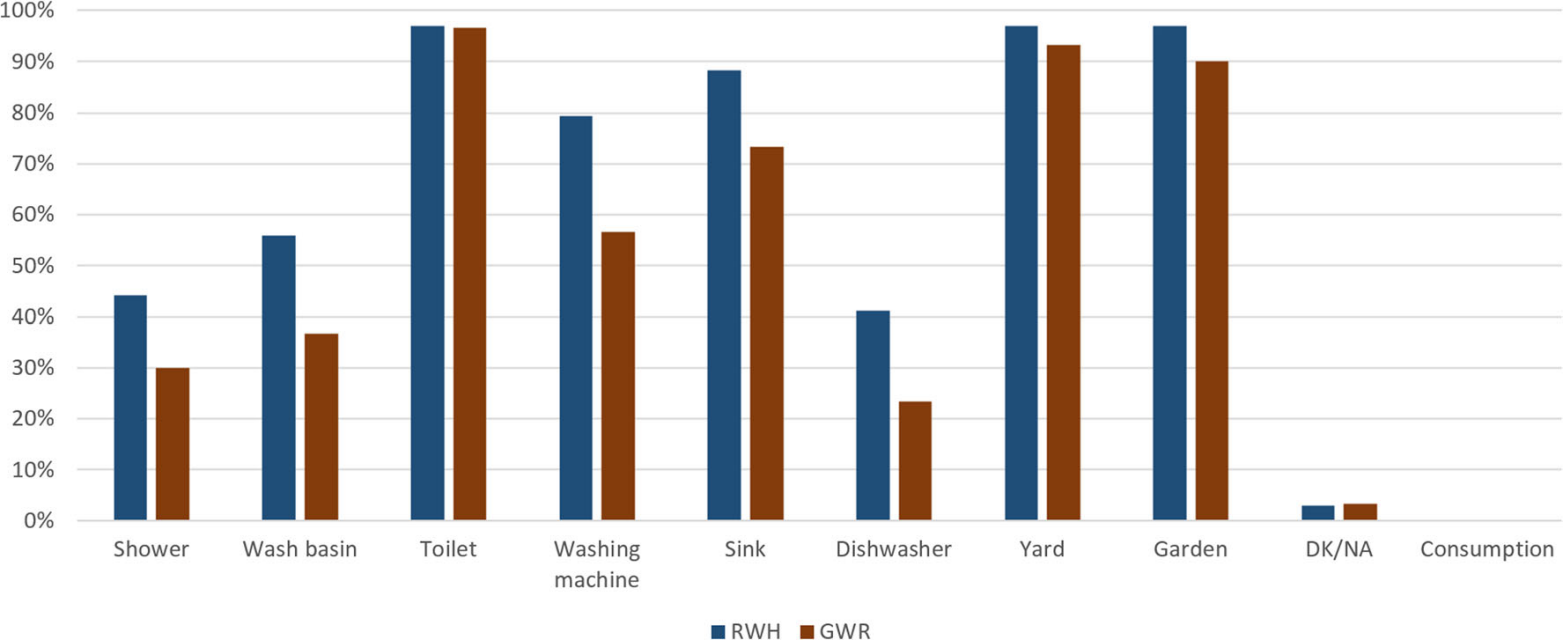
End-uses in which participants were willing to utilise rainwater harvesting or greywater reuse

Participants expressed greater willingness to use GWR and RWH in areas of the home including toilets, patio, garden, laundry and washing machine (Fig. 1), coinciding with the accepted uses in countries such as South Africa (Dobrowksy et al. 2014), the UK (Ward et al. 2013) and Oman (Prathapar et al. 2005). Based on these results, it was decided to include these end-uses as those proposed to be connected to the designed AWSS due to the demonstrated acceptability.

Regarding the willingness of users to perform reactive maintenance on the AWSS, 94% of participants indicated that they would be willing to carry out this type of activity once every 2 weeks, 83% once a week, 34% twice a week and only 9% daily. Concerning preventive maintenance, 94% of participants suggested that they would perform this maintenance annually, 83% every 6 months and 37% monthly. Taking into account the availability of householders to perform maintenance tasks is an integral element in designing the system and is of importance as it is one of the factors affecting the acceptance of such systems, as identified in the UK by Ward et al. (2013).

In relation to the willingness to pay for implementing RWH and GWR systems in a new household, participants would be willing to increase the initial investment cost compared to that of a conventional household as follows: (a) less than 2300 USD, 94% of participants; (b) between 2300 and 4900 USD, 54%; and (c) more than 4900 USD, 14%. These findings excluded the consideration of some GWR proposals in this study, for instance, prefabricated or proprietary off-the-shelf devices that met drinking water quality standards, whose costs for 2015 were 15,800 USD and which are available from Latin American providers (Agua2use 2015).

#### Water quality for RWH and GWR systems

A review of literature on the physicochemical and microbiological parameters in RWH and GWR systems was conducted in order to incorporate the information into the selection and design of the treatment system, primarily relating to concerns raised in the questionnaire results. The review focused on parameters such as pH, total suspended solids (TSSs), turbidity, heavy metals, total and faecal coliforms. The search was conducted in specialised journals from databases such as Scopus®, ScienceDirect®, CRCnetdatabase®, EBSCO® and Springer®.

According to research conducted in Israel (Friedler 2004), Portugal (Matos et al. 2015), UK (ETH 2009) and other countries (Ghaitidak and Yadav 2013), it was identified that the shower is the sanitary device with better physicochemical characteristics for GWR, and therefore, it was selected as the source of greywater in this study.

Harvested rainwater quality, however, depends on the number of dry days before precipitation (Kwaadsteniet et al. 2013); roof material (Lee et al. 2012); building design features (Ward et al. 2010); and external contamination from roofs, canals and air pollution (Mendez et al. 2011), which can generate significant concentrations of ions (ammonium, nitrate and sulphate), especially in highly urbanised areas (Sánchez et al. 2015). Table 1 compiles the results from nine investigations about rainwater quality in urban environments in different countries, with monthly average rainfall greater than 60 mm, and compares them against three standards for reuse in potable and non-potable water uses. According to the information reported in Table 1, national quality standards for the use of rainwater in non-potable purposes in some countries are pH, TSS, turbidity, CT and *Escherichia coli*. Additionally, the studies that have evaluated the quality of harvested rainwater in different contexts indicate that generally, the parameters associated to this water source exceed the limit values set by those standards. Consequently, consideration of treatment processes and decisions regarding whether or not to include them may be based on site factors, human factors or, less so, on empirical data collected at a proposed site. The authors could not identify any water quality data for the specific case study site in Colombia focused on in the present research; therefore, this brief review suggests that the inclusion of contamination prevention or treatment systems for RWH and GWR should be considered at the design stage, in order to minimise contamination mainly associated with TSS, turbidity and total and faecal coliforms.

**Table 1:** Studies involving harvested rainwater quality parameters in urban areas with average monthly rainfall greater than 60 mm compared with different national quality standards for potable and non-potable use

Country	Material	Samples	pH (mg/L)	TSS (mg/L)	Turbidity (NTU)	Fe (μg/L)	Pb (μg/L)	Zn (μg/L)	NO_3_ (mg/L)	CT (CFU/100 mL)	*E. coli* (CFU/100 mL)	Reference
Colombia	c	2	7.1	38	6	177	–	–	–	–	–	Estupiñán et al. (2011)
Colombia	c	2	7.6	–	6	24	0.2	–	–	–	–	
Texas, USA	ct	4	7.7	140	51	950	3.5	160	3.3	1500	–	Mendez et al. (2011)
Greece	c	156	8.3	–	–	11	0.2	10	7.1	11	0	Sazakli (2007)
South Korea	c	90	7.3	–	–	–	27	160	2.2	70	10	Lee et al. (2010)
South Korea	ct	40	7.1	219	–	155	11.0	131	1.9	76	8	Lee et al. (2012)
Bermuda	∼	112	7.8	112	–	8	0.2	9	1.6	–	–	Peters et al. (2008)
West Australia	∼	15–70	6.7	6	1	44.8	3.8	1790	1.2	100	0	CRC AUS (2008)
East Australia	∼	165–354	6.1	–	–	68	5.4	770	1.6	–	–	Husto et al. (2012)
England, UK	∼	40	7.6–10.4	–	0.3–2.8	9–27.4	25.5–64.4	193–480	1.32–17.74	0–2600	–	Ward et al. (2010)
Range	–	–	6.1–10.4	6–219	0.3–51	8–950	0.2–64.4	9–1790	1.32–17.74	0–2600	8–10	–
Australia^a^	Non-potable use		6.5–8.5	<10	<2–5	–	–	–	–	<1	–	GWA (2011)
USA^a^	Potable use		6.5–8.5	0	<1	300	0	5000	10	0	0	USEPA 2009
UK^a^	Non-potable use		5–9.5	–	<10	–	–	–	–	1000	250	BSI (2013)

*∼* Various materials, *–* not specified, *c* concrete, *ct* clay tile

^a^Quality parameters of various regulations for potable and non-potables uses

### Formulation and assessment of alternative system designs for RWH and GWR

#### Proposed alternatives

**Fig. 2:**
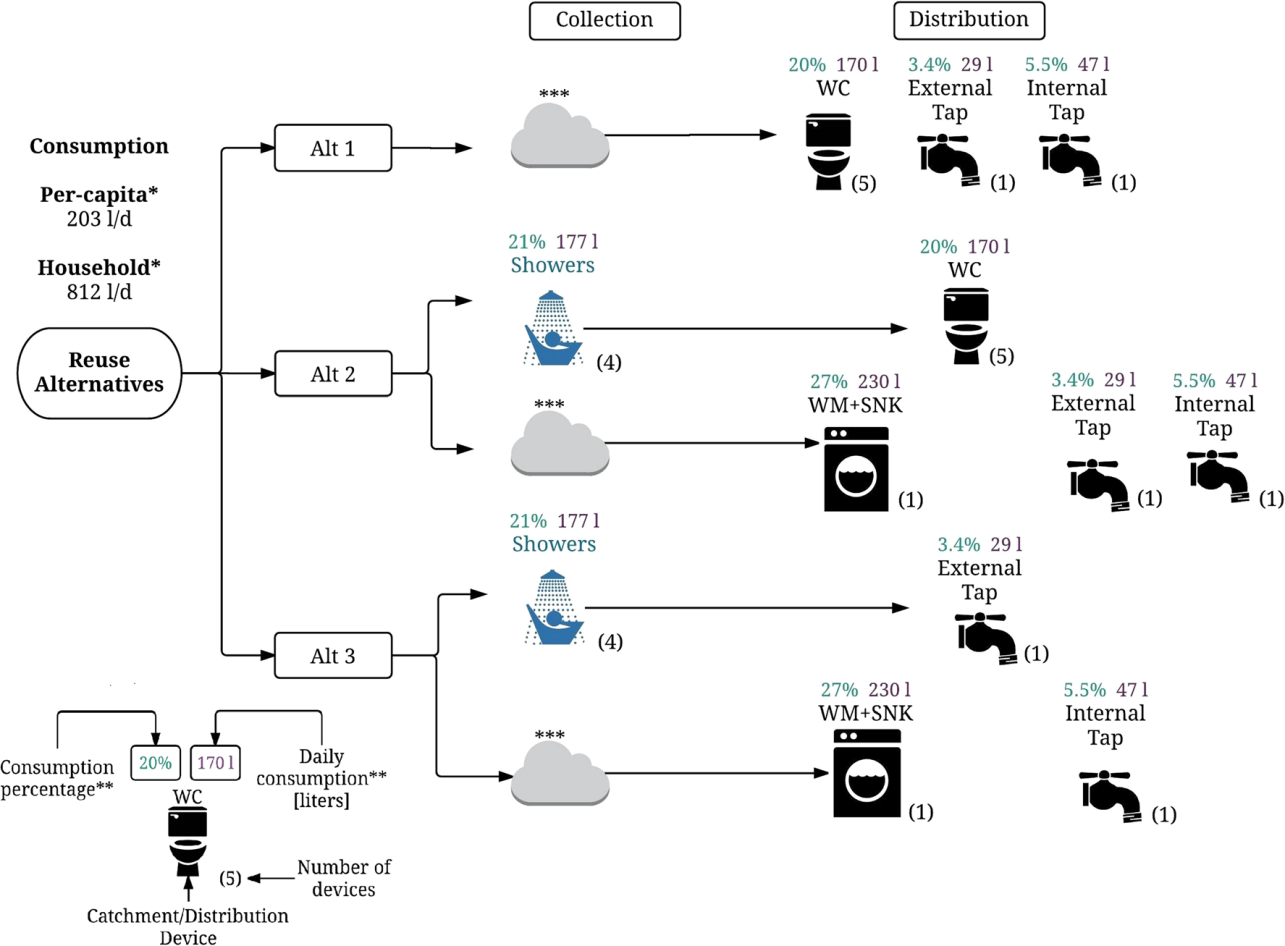
Three proposed alternative rainwater harvesting and greywater reuse systems detailing water sources and potential end-uses

Three alternatives (Alt 1, Alt 2 and Alt 3), summarised in Fig. 2, for the GWR and RWH systems were initially proposed trying to balance end-user preferences and water availability. For this, a factor that weights two features was computed: (i) users’ preferences concerning end-uses for the alternative sources (rainwater and greywater), captured through the household survey (users’ acceptability (u.a.)), and (ii) water demand for each end-use. The weight of the features was defined based on the authors’ experience, where the u.a. had greater weight (0.8) than end-use water demand (0.2), as it is well documented that acceptability can limit utilisation of alternative water supplies rather than availability. Table 2 summarises the results for the calculation of this factor (the potential end-use factor (p.eu.f)).

**Table 2:** Potential end-use factor

Factors	sh	wb	to	wm	snk	dw	it^d^	et^d^
u.a.rw^a^	0.44	0.56	0.97	0.79	0.88	0.41	0.97	0.97
u.a.gw^a^	0.30	0.37	0.97	0.57	0.73	0.23	0.93	0.90
Consumption^b^	0.21	0.04	0.20	0.17	0.10	0.16	0.06	0.03
p.eu.f.rw^c^	0.39	0.46	0.82	0.67	0.72	0.36	0.79	0.78
p.eu.f.gw^c^	0.32	0.39	0.94	0.59	0.73	0.26	0.90	0.88

^a^u.a.() user acceptability scaled from 0 to 1 for rainwater (rw) and greywater (gw), based on survey results

^b^Indicated as percentage of total household consumption, based on CRA (2001)

^c^p.eu.f.() potential end-use factor = [u.a.() × 0.8] + [consumption × 0.2]

^d^(sh) shower, (wb) wash basin, (to) toilet, (wm) washing machine, sink (snk), dishwashing (dw), (it) interior tap, (et) external tap (i.e. garden). End-uses it and et are for cleaning and irrigation only

Using results of the potential end-use factor for rainwater (p.eu.f.rw) and greywater (p.eu.f.gw), Alt 1 consisted of harvested rainwater collection, treatment and storage for use in toilets (0.82 p.eu.f.rw ), external tap (0.79 p.eu.f.rw) and internal taps (0.78 p.eu.f.rw) (i.e. the three higher p.eu.f.rw). Alt 2 consisted of harvested rainwater for use in washing machines (0.67 p.eu.f.rw), sinks (0.72 p.eu.f.rw), internal taps (0.79 p.eu.f.rw) and external taps (0.78 p.eu.f.rw) and greywater from showers for use in toilets (0.94 p.eu.f.gw) (i.e. the five higher p.eu.f.gw). Alt 3 consisted of harvested rainwater for use in washing machines (0.67 p.eu.f.rw), sinks (0.72 p.eu.f.rw) and internal taps (0.79 p.eu.f.rw) and greywater from showers for use in external taps (0.88 p.eu.f.gw). Since Colombia lacks regulation of AWSS, usage in sinks is considered as a non-drinking water residential use, despite the possibility that water from sink taps may be ingested.

For each of these alternatives, given in the following sections, an initial analysis of the savings in water and energy demand was made. The alternative that offered greater water savings and lower energy costs was assessed further based on additional criteria such as network configuration, which is described in the “Design of RWH and GWR system alternative 2” section.

#### Dimensioning of storage tanks

## Rainwater

The method proposed in this study consisted of assessing five options for the tank capacity (*T*_1_ 1 m^3^, *T*_2_ 2 m^3^, *T*_3_ 3 m^3^, *T*_4_ 4 m^3^, *T*_5_ 5 m^3^), considering the commercially available tanks in Colombia and the supply from harvested rainwater that could be available, established from rainfall records for a 15-year period (1985–2001). Daily rainfall records were obtained from a meteorological station located 2 km from the study area and operated by the Instituto de Hidrología, Meteorología y Estudios Ambientales from Colombia (IDEAM). The estimation was conducted as follows:

i) The daily rainfall needed to fill each tank size (*P*_*Ti*_) was estimated with the available roof area (*A*) (101 m^2^). As result, *P*_*Ti*_ values for each tank were *P*_*T*1_ 9.90 mm, *P*_*T*2_ 18.80 mm, *P*_*T*3_ 29.70 mm, *P*_*T*4_ 39.60 mm and *P*_*T*5_ 49.51 mm.
ii) The volume of harvested rainwater was estimated for each week and each tank size considering
  Rainfall episodes each week where precipitation was lower than *P*_*Ti*_.
  Rainfall episodes each week where precipitation was equal or higher than *P*_*Ti*_.

The rainfall episodes each week where precipitation was lower than *P*_*Ti*_ were added to establish a cumulative value (*L*). For the rainfall episodes each week, where precipitation was equal or higher than *P*_*Ti*_, the number of days this situation occurred was determined each week (*F*). It was considered that overflows during these days would enter the drainage system (e.g. for *T*_1_, 10.30 mm rain would produce an overflow of 10.30 − 9.90 mm = 0.40 mm). Equation 1 indicates the formula used to establish the volume of harvested rainwater for each month and each tank size option,

1 $$ {V}_{i, j- Ti}=\left[\left(\sum L\times C\right)+\left( F\times {P}_{Ti}\right)\right]\times \frac{A}{1000\kern0.5em \mathrm{mm}/{\mathrm{m}}^3} $$

where *V*_*i,j* − *Ti*_ is the water volume cumulated in week *i*, from year *j*, for a storage tank of size *i* (m^3^); *L* is the rainfall values in week *i*, for which daily rainfall was less than *P*_*Ti*_ (mm); *C* is the runoff coefficient for the roof area (in this case, 0.9 as recommended for clay tiles; UNATSABAR 2001); *F* is the number of days in week *i*, with rainfall higher than *P*_*Ti*_; *P*_*Ti*_ is the rainfall needed to fill the storage tank *i* (mm); and *A* is the roof area (m^2^) (in this case, 101 m^2^).

The average weekly volume of rainwater harvested for each tank of the analysed sizes was calculated with the values established for each year obtained as shown in Eq. 1 (values for 15 years). The annual supply of harvested rainwater was determined as the total of the average water volumes calculated for each week of the year (see Table 3). Finally, water volumes from the rainwater harvested during a year in tanks of consecutive sizes were compared as shown in Table 3.

**Table 3:** Annual rainwater available for various tank sizes

Week	Units	Storage tank size				
		*T*_1_	*T*_2_	*T*_3_	*T*_4_	*T*_5_
1	m^3^/week	0.83	1.00	1.11	1.17	1.21
2	m^3^/week	0.79	1.16	1.30	1.36	1.40
3	m^3^/week	0.60	0.75	0.82	0.86	0.86
4	m^3^/week	0.58	0.74	0.84	0.86	0.86
5	m^3^/week	0.88	1.24	1.39	1.46	1.48
6	m^3^/week	1.10	1.46	1.59	1.59	1.59
7	m^3^/week	0.41	0.47	0.53	0.53	0.53
8	m^3^/week	1.01	1.51	1.86	2.10	2.22
9	m^3^/week	1.21	1.33	1.38	1.38	1.38
10	m^3^/week	0.89	1.22	1.45	1.59	1.61
11	m^3^/week	1.23	1.51	1.65	1.74	1.74
12	m^3^/week	1.22	1.51	1.62	1.62	1.62
13	m^3^/week	1.64	2.40	2.84	3.12	3.24
14	m^3^/week	0.95	1.41	1.61	1.71	1.77
15	m^3^/week	0.86	1.24	1.35	1.40	1.40
16	m^3^/week	1.56	2.04	2.28	2.44	2.54
17	m^3^/week	1.52	2.03	2.22	2.35	2.40
18	m^3^/week	1.37	1.73	1.81	1.87	1.93
19	m^3^/week	1.21	1.56	1.75	1.81	1.85
20	m^3^/week	0.95	1.10	1.23	1.29	1.36
21	m^3^/week	2.10	2.66	2.84	2.94	2.97
22	m^3^/week	1.30	1.68	1.94	2.08	2.20
23	m^3^/week	1.00	1.26	1.37	1.42	1.42
24	m^3^/week	1.08	1.37	1.49	1.52	1.52
25	m^3^/week	1.18	1.28	1.28	1.28	1.28
26	m^3^/week	1.04	1.23	1.26	1.26	1.26
27	m^3^/week	1.53	1.74	1.83	1.89	1.95
28	m^3^/week	1.30	1.64	1.69	1.69	1.69
29	m^3^/week	1.48	1.88	2.07	2.25	2.36
30	m^3^/week	1.36	1.87	2.06	2.13	2.13
31	m^3^/week	1.03	1.21	1.34	1.44	1.44
32	m^3^/week	1.21	1.37	1.43	1.43	1.43
33	m^3^/week	1.07	1.33	1.48	1.61	1.73
34	m^3^/week	0.94	1.00	1.00	1.00	1.00
35	m^3^/week	1.60	1.93	2.15	2.22	2.28
36	m^3^/week	1.38	1.62	1.69	1.69	1.69
37	m^3^/week	1.67	1.99	2.09	2.15	2.22
38	m^3^/week	1.56	2.09	2.38	2.50	2.56
39	m^3^/week	1.55	1.91	2.00	2.06	2.12
40	m^3^/week	1.56	2.08	2.34	2.48	2.56
41	m^3^/week	1.99	2.66	2.77	2.83	2.85
42	m^3^/week	1.43	1.90	2.21	2.34	2.46
43	m^3^/week	1.20	1.56	1.82	2.00	2.12
44	m^3^/week	1.18	1.44	1.67	1.80	1.89
45	m^3^/week	1.35	1.79	2.04	2.23	2.32
46	m^3^/week	1.04	1.28	1.32	1.32	1.32
47	m^3^/week	1.42	1.80	1.93	1.97	1.97
48	m^3^/week	0.92	1.16	1.31	1.44	1.54
49	m^3^/week	0.96	1.13	1.14	1.14	1.14
50	m^3^/week	0.70	0.85	0.96	0.96	0.96
51	m^3^/week	0.32	0.40	0.44	0.44	0.44
52	m^3^/week	0.50	0.56	0.56	0.56	0.56
Total year	m^3^/year	60.78	77.11	84.53	88.31	90.37
Difference	m^3^	–	16.33	7.42	3.78	2.06
Increase	%	–	26.86	9.62	4.47	2.33

Although a bespoke methodology was used to determine the size of the tank, the works of Sanches Fernandes et al. (2015), Santos and Taveira-Pinto (2013), Ghisi (2001) and Fewkes and Butler (2000) were taken into account to conduct the sizing.

The difference in water volumes that could be stored comparing tanks of consecutive sizes 1, 2 and 3 m^3^ was considerable, and from the tank of 4 m^3^, the savings differential reduced. For this reason, tanks *T*_2_ and *T*_3_ (i.e. 2 and 3 m^3^, respectively) were considered as potential options to implement. The analysis of supply and demand is presented in the “Initial assessment of alternatives” section.

## Greywater

Tank dimensioning for the GWR system considered the use frequency of the devices that contributed to greywater supply and demand (Fig. 2). As greywater cannot remain in the tank for more than 48 h (Al-Jayyousi 2003; March and Gual 2009) due to the exponential growth of microorganisms, the volume of storage required (*V*_required_) must be equal to the volume of demand in 48 h (*V*_demand_ in a cycle of use) to ensure (i) water supply and (ii) the renewal of water.

In Alt 2, water supply and water demand were considered at a daily frequency. Thus, the required storage was the smallest value between demand (169 L/day) and supply (177 L/day) (Eq. 2).

2 $$ {V}_{\mathrm{required}}=\left({V}_{\mathrm{daily}\ \mathrm{offer}},\kern0.5em {V}_{\mathrm{daily}\ \mathrm{demand}}\right) $$

For Alt 3, the household survey indicated that the use of the garden tap was once every 3 days (2.5 times per week), for a demand of 83 L/use. The water used in showers (daily use) provided a volume of 177 L/day. Thus, the required volume of the tank was 83 L, but the selected tank was 300-L capacity due to the sizes available commercially and considering the installation of a drain when the water level corresponds to a volume of 90 L to ensure the periodic renewal of the stored greywater.

### Initial assessment of alternatives

Using the outline alternative system configurations and tank sizes, drinking water savings and energy consumption were assessed in order to select one of the three alternative system configurations for a full financial analysis. As the three configurations had similar social acceptability and technical requirements, it was decided to focus on complete evaluation of the configuration with the greater savings and lower-energy implications.

## Drinking water savings

Drinking water savings due to RWH were computed based on the difference between weekly supply and demand, adding accumulations from previous week (if generated). Supply was considered variable and estimated as explained in the “Dimensioning of storage tanks” section, whilst demand was assumed constant. The procedure was as follows:

i) Demand was established considering the proportion of water consumed by each end-use (CRA 2001), 19.9% toilets, 17.0% washing machine, 10.2% sink, 3.4% external tap and 5.5% internal tap. The water quantity for each of the proposed alternatives, considering the total water consumption in the household (847.51 L/day) and the consumption in the selected end-uses, was 244 L/day (Alt 1), 259 L/day (Alt 2) and 277 L/day (Alt 3).
ii) Demand was established for each week of the year, for each alternative, 1709 L/week (Alt 1), 2141 L/week (Alt 2) and 1939 L/week (Alt 3).
iii) Weekly supply was estimated for each week, based on the calculations described in the “Dimensioning of storage tanks” section (according to Table 3).
iv) The difference between supply and demand was calculated for each week of the year, in each year between 1986 and 2001.
v) When the difference between supply and demand was positive, the exceeding volume was added to the supply for the following week, unless it exceeded the tank volume, in which case it was allocated as overflow to drainage. When the difference between supply and demand was negative, supply was considered insufficient for the demand in the tank option (*T*_*i*_) and alternative assessed (Alt_X_).
vi) Drinking water savings due to RW were established considering the higher value between supply and demand for each week. This was calculated for each year and each alternative. It was considered that if at the end of a week there was rainwater surplus, only an amount equal to or less to the tank volume could be stored.
vii) Drinking water savings were estimated for each year (1986 to 2001), and finally, an average of the annual water savings of each alternative was calculated (see Table 4).

**Table 4:** Drinking water annual savings (volume and costs) for three Alt RWH system configurations

Week	Unit	Alt 1		Alt 2		Alt 3	
		*T*_2_	*T*_3_	*T*_2_	*T*_3_	*T*_2_	*T*_3_
1	m^3^/week	0.8	0.8	0.9	0.9	0.9	0.9
2	m^3^/week	0.8	0.8	0.9	0.9	0.9	0.9
3	m^3^/week	0.9	0.9	0.9	1.0	0.9	0.9
4	m^3^/week	0.6	0.8	0.6	0.8	0.6	0.8
5	m^3^/week	1.0	1.0	1.1	1.1	1.1	1.1
6	m^3^/week	1.1	1.1	1.2	1.3	1.2	1.2
7	m^3^/week	0.9	0.9	0.8	0.9	0.8	0.9
8	m^3^/week	0.9	1.0	1.0	1.0	0.9	1.0
9	m^3^/week	1.2	1.2	1.4	1.4	1.3	1.3
10	m^3^/week	1.2	1.2	1.2	1.3	1.2	1.3
11	m^3^/week	1.5	1.5	1.5	1.7	1.5	1.6
12	m^3^/week	1.1	1.3	1.2	1.3	1.1	1.3
13	m^3^/week	1.4	1.4	1.6	1.6	1.5	1.5
14	m^3^/week	1.4	1.4	1.6	1.7	1.5	1.5
15	m^3^/week	1.2	1.3	1.2	1.4	1.2	1.4
16	m^3^/week	1.6	1.6	1.9	1.9	1.7	1.8
17	m^3^/week	1.5	1.6	1.6	1.8	1.6	1.8
18	m^3^/week	1.7	1.7	1.8	2.0	1.8	1.9
19	m^3^/week	1.6	1.7	1.7	1.9	1.6	1.8
20	m^3^/week	1.3	1.6	1.3	1.3	1.3	1.4
21	m^3^/week	1.6	1.6	1.8	1.9	1.7	1.8
22	m^3^/week	1.5	1.5	1.7	1.8	1.6	1.7
23	m^3^/week	1.3	1.6	1.3	1.6	1.4	1.6
24	m^3^/week	1.1	1.2	1.2	1.2	1.1	1.2
25	m^3^/week	1.2	1.2	1.2	1.2	1.2	1.3
26	m^3^/week	1.1	1.2	1.1	1.2	1.1	1.2
27	m^3^/week	1.4	1.4	1.5	1.5	1.4	1.5
28	m^3^/week	1.5	1.5	1.7	1.7	1.6	1.6
29	m^3^/week	1.4	1.4	1.5	1.6	1.5	1.5
30	m^3^/week	1.4	1.4	1.6	1.6	1.5	1.5
31	m^3^/week	1.2	1.3	1.3	1.4	1.3	1.3
32	m^3^/week	1.4	1.4	1.4	1.6	1.5	1.5
33	m^3^/week	1.2	1.3	1.1	1.3	1.1	1.4
34	m^3^/week	1.3	1.5	1.3	1.4	1.3	1.4
35	m^3^/week	1.4	1.4	1.6	1.6	1.5	1.5
36	m^3^/week	1.5	1.5	1.6	1.7	1.7	1.7
37	m^3^/week	1.4	1.5	1.5	1.6	1.4	1.6
38	m^3^/week	1.6	1.7	1.8	1.9	1.7	1.7
39	m^3^/week	1.5	1.6	1.7	1.7	1.6	1.6
40	m^3^/week	1.6	1.6	1.9	2.0	1.8	1.8
41	m^3^/week	1.5	1.6	1.6	1.8	1.5	1.7
42	m^3^/week	1.5	1.6	1.8	1.8	1.7	1.7
43	m^3^/week	1.3	1.5	1.4	1.6	1.4	1.5
44	m^3^/week	1.2	1.3	1.3	1.5	1.3	1.4
45	m^3^/week	1.4	1.4	1.6	1.7	1.5	1.6
46	m^3^/week	1.3	1.4	1.3	1.5	1.3	1.5
47	m^3^/week	1.4	1.5	1.5	1.6	1.4	1.5
48	m^3^/week	1.3	1.4	1.2	1.4	1.3	1.4
49	m^3^/week	1.2	1.4	1.3	1.4	1.2	1.3
50	m^3^/week	1.1	1.3	1.0	1.2	1.1	1.2
51	m^3^/week	0.6	0.7	0.5	0.6	0.5	0.7
52	m^3^/week	0.5	0.7	0.5	0.6	0.5	0.6
Total year	m^3^/year	65.6	69.5	70.6	75.3	68.7	73.1
Costs saved^a^	[USD]/year	49.8	52.8	53.7	57.2	52.2	55.5
Difference	[USD]/year	3.0		3.6		3.3	

^a^Estimated based on the water tariff for year 2015 of 0.76 USD/m^3^ (amb 2015)

Table 4 includes the annual volume of drinking water saved and the associated costs due to the storage tanks of 2 m^3^ (*T*_2_) and 3 m^3^ (*T*_3_) for Alt 1, Alt 2 and Alt 3. System efficiency was estimated considering the annual water savings obtained from each alternative and the annual demand of the system. The following results were obtained: Alt 1 77.2% (*T*_2_) and 78.2% (*T*_3_), Alt 2 63.4% (*T*_2_) and 67.6% (*T*_3_) and Alt 3 68.1% (*T*_2_) and 72.4% (*T*_3_).

The difference in volume of drinking water saved by using a tank of 3 m^3^ or a tank of 2 m^3^ (i.e. between 0.90 and 4.72 m^3^) was considered low compared to the higher acquisition, energy and construction costs associated with the tank of 3 m^3^. Therefore, it was decided to use a RWH system with a storage tank of 2 m^3^ in the final design configuration.

In the case of the GWR system, supply and demand were considered constant during the year. Therefore, the drinking water saving in this system was the lower value between supply and demand; therefore, the volume of drinking water saved during a year due to GWR in each alternative was (i) 0.00 m^3^, (ii) 60.72 m^3^ and (iii) 10.37 m^3^.

## Energy consumption

The energy consumption of the RWH and GWR system was due to the pumps required to deliver stored water into the pipe network of the house. To establish this consumption, the power of the pump (*P*_pump_) and operating time (*t*) required estimation. *t* depended on the volume of water to be pumped (vol) and pump flow (*Q*_pump_), which must be greater than the supply flow needed (i.e. determined by the Hunter method for the devices where greywater will be reused). The procedure is summarised below (Eqs. 3 and 4),

3 $$ t\left[ h\right]=\frac{\mathrm{vol}\left[{\mathrm{m}}^3\right]}{Q_{\mathrm{pump}}\left[\raisebox{1ex}{${\mathrm{m}}^3$}\!\left/ \!\raisebox{-1ex}{$\mathrm{h}$}\right.\right]} $$

4 $$ \mathrm{Energy}\ \mathrm{consumption}\left[\mathrm{kWh}\right]={P}_{\mathrm{pump}}\left[ h p\right]\times 0.746\times t\left[ h\right] $$

The operationally derived method outlined in Ward et al. (2011) was not suitable for application in this case due to the system under consideration being hypothetical rather than implemented.

Concerning the parameters for the RWH and GW pumps, the following conditions were established:

- GW pump net positive suction head (NPSH_req)_ 7.072 m, hydraulic power (hhp) 0.058 hp, required power 0.269 hp and nominal power 0.5 hp.
- RW pump NPSH_req_ 7.080 m, hhp 0.058 hp, required power 0.135 hp and nominal power 0.25 hp.

The results obtained by applying Eqs. 3 and 4 are summarised in Table 5. The required power and nominal power of the pumps for GWR and RWH in the three alternatives are presented. Alt 2 had the highest energy consumption (20.6 kW in a year), as in this alternative, a greater amount of water must be pumped. However, this energy consumption was lower compared to that of a conventional fan (200 W/h), working 6 h a day per month (432 kW in a year).

**Table 5:** Energy consumption for three alternative RWH and GWR system configurations

Alternative		Unit	Alt 1	Alt 2	Alt 3
RWH	Utilization		to, et, it	snk, wm, et, it	snk, wm, it
	Volume^a^	m^3^	65.6	70.6	68.7
	Flow rate	L/s	0.68	0.37	0.43
	Required power	HP	0.31	0.13	0.16
	Nominal power	HP	0.50	0.25	0.25
	Time	h/year	27	53	44
	kW/year^b^	kW	10.0	9.94	8.27
GWR	Reuse		–	to	et
	Volume^a^	m^3^	–	60.72	10.37
	Flow rate	L/s	–	0.59	0.20
	Required power	HP	–	0.47	0.09
	Nominal power	HP	0	0.50	0.25
	Time	h/year	0.00	29	14
	kW/year^b^	kW	0	10.66	2.69
Total	kW/year^b^		10.0	20.6	11.0

*to* toilet, *et* external tap, *it* interior tap, *snk* sink, *wm* washing machine

^a^m^3^

^b^kW

## Selection of an alternative RWH and GWR configuration

Table 6 presents a comparison of the three alternative RWH and GWR system configurations against criteria for drinking water and energy savings. In addition to the drinking water and energy savings estimated in the previous sections, Table 6 includes the cost savings associated with reduced drinking water consumption, energy consumption and sewerage reduction based on rates provided by different utility companies (amb 2015 for drinking water; EMPAS 2015 for sewerage and ESSA 2015 for electricity). These were estimated using simple calculations based on the number of units saved multiplied by the value of a unit. After comparing the results for these criteria, Alt 2 was selected as the RWH and GWR systems to assess further due to the greater estimated potential financial savings (∼166.1 USD/year).

**Table 6:** Summary of selection criteria to initially assess three alternative RWH and GWR system designs

Criteria		Unit	Alternative		
			Alt 1	Alt 2	Alt 3
RWH	Drinking water saved	m^3^/year	65.6	70.6	68.7
	Energy consumption	kWh/year	10.0	9.9	8.3
	Drinking water cost	USD/year	49.8	53.7	52.2
	Sewerage cost	USD/year	31.5	33.9	33.0
	Energy cost	USD/year	1.6	1.6	1.3
GWR	Drinking water saved	m^3^/year	–	60.7	10.4
	Energy consumption	kWh/year	–	10.6	2.7
	Drinking water cost	USD/year	–	46.1	7.9
	Sewerage cost	USD/year	–	29.2	5.0
	Energy cost	USD/year	–	1.7	0.4
Total	USD		82.9	166.1	99.8

Calculations made with the following rates: water supply = 0.76 USD/m^3^ (amb 2015), sewerage = 0.48 USD/m^3^ (EMPAS 2015) and energy = 0.16 USD/kWh (ESSA 2015)

### Design of RWH and GWR system alternative 2

To further assess the financial feasibility of the Alt 2 RWH and GWR systems, additional criteria were considered, including the network layout of the different supply systems involved in the overall residential water supply system and the treatment systems required for each source water.

#### Collection and distribution network

The full design of Alt 2 considered the different types of networks required in a residential building, which are drinking water (DW), wastewater (WW), collected greywater (CGW), treated greywater (TGW), collected rainwater (CRW) and treated rainwater (TRW). The networks’ layout was designed based on concepts from detailed guidelines for domestic water systems (Woolley 2002) and Colombian regulations—NTC 1500 (ICONTEC 2004). Autodesk Revit® software was used, as this enables a workflow-type Building Information Modelling (‘BIM’) approach that includes the consideration of architectural and structural engineering elements within the designs. In addition, a hydro-pneumatic system design for each of the reuse networks (i.e. TGW and TRW) was included.

The layout and hydraulic networks were designed considering flexibility. Thus, if required, the system could be manually disconnected from the internal distribution network, and all devices could be supplied with drinking water. To enable this, automatic electro-mechanical valves and electric floats, controlled by the end-user or by the tank level, were incorporated.

**Fig. 3:**
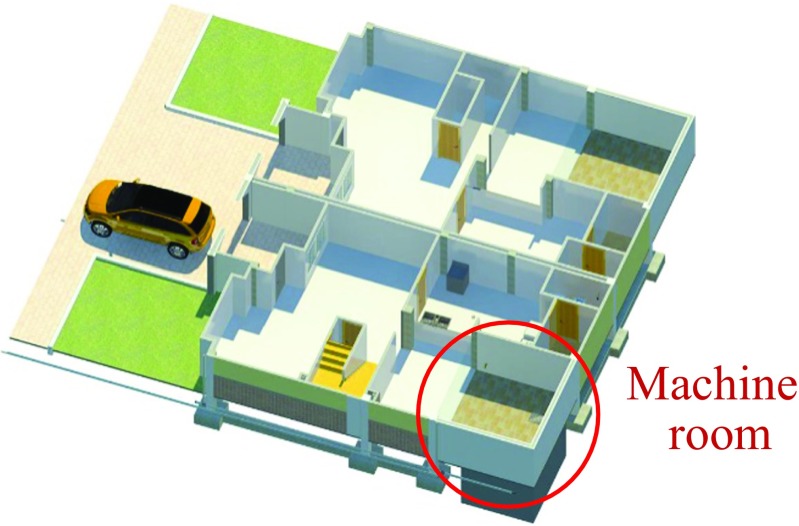
Machine room location

**Fig. 4:**
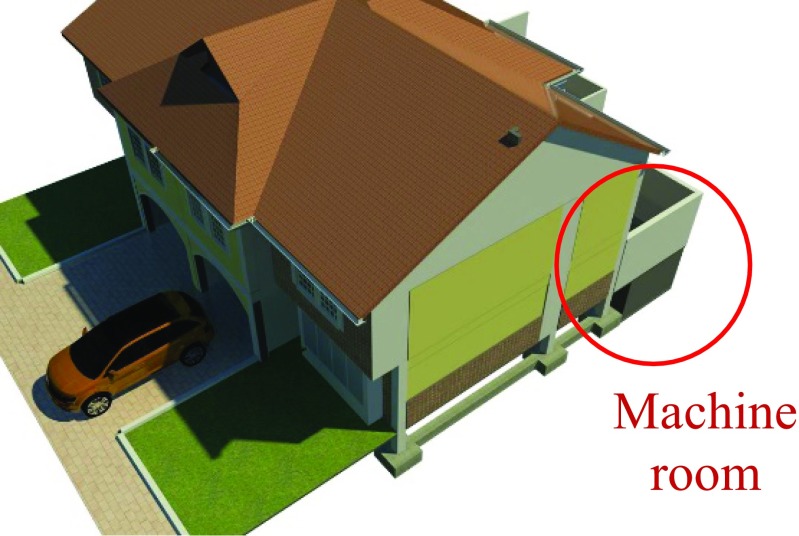
Exterior 3D view of the machine room location

Within the existing residential building design, the RWH and GWR system’s machine room was located below the courtyard garden because of its easy access for maintenance and the possibility of gravity collection of greywater and rainwater without affecting the original architectural design. The disadvantage of this location was the need for excavation and construction of retaining walls in reinforced concrete (Figs. 3 and 4).

### Treatment system

## Rainwater

The RWH system design included a leaf filter and gutter guards to prevent the entry of insects, particulate matter and organic contaminants (OPS 2004). Additionally, it considered a first flush diversion device to improve the quality of the captured water by separating the flow of the first millimetres of rain, where water is more polluted (Sánchez et al. 2015). Diverting this first flush minimises the physicochemical contamination, but additional components must be included for microbiological treatment (Gikas and Tsihrintzis 2012), and remotion of remaining turbidity, ions (i.e. Al, Fe) and total coliforms, since these elements can generate unwanted odours and water discoloration (Mendez et al. 2011). Additionally, a self-cleaning filter that reduces pollution by heavy metals and improves levels of turbidity and TSS was included. This filter has adjustable treatment capabilities, does not consume energy, maintenance is performed in periods longer than 2 years and it can be built with locally available materials (Vieira et al. 2013).

In general, the described treatment ensures compliance with quality requirements to store and distribute rainwater for non-drinking domestic uses (ARCSA-ASPE 2012; Sanches Fernandes et al. 2015; Texas WDB 2005). However, in cases where there are birds in the area, compliance with microbiological parameters may require disinfection treatment after storage (Lee et al. 2010), such as with a UV device.

## Greywater

Table 7 characterises the levels and treatment technologies usually implemented for GWR.

**Table 7:** Most used greywater treatment technologies

Type	Remove	Processes
Preliminary	Fats, hairs and suspended particles	Solid and fat removal and filtration
Primary	Settleables and suspended solids	Sedimentation and filtration
Secondary	Biodegradable matter and heavy metals	Filtration, biodegradation and adsorption
Tertiary	Nutrients and microbiological agents	Disinfection, nano-filtration and ion exchange

*Sources*: Ghaitidak and Yadav (2013), Merz et al. (2007) and Pidou et al. (2007)

**Fig. 5:**
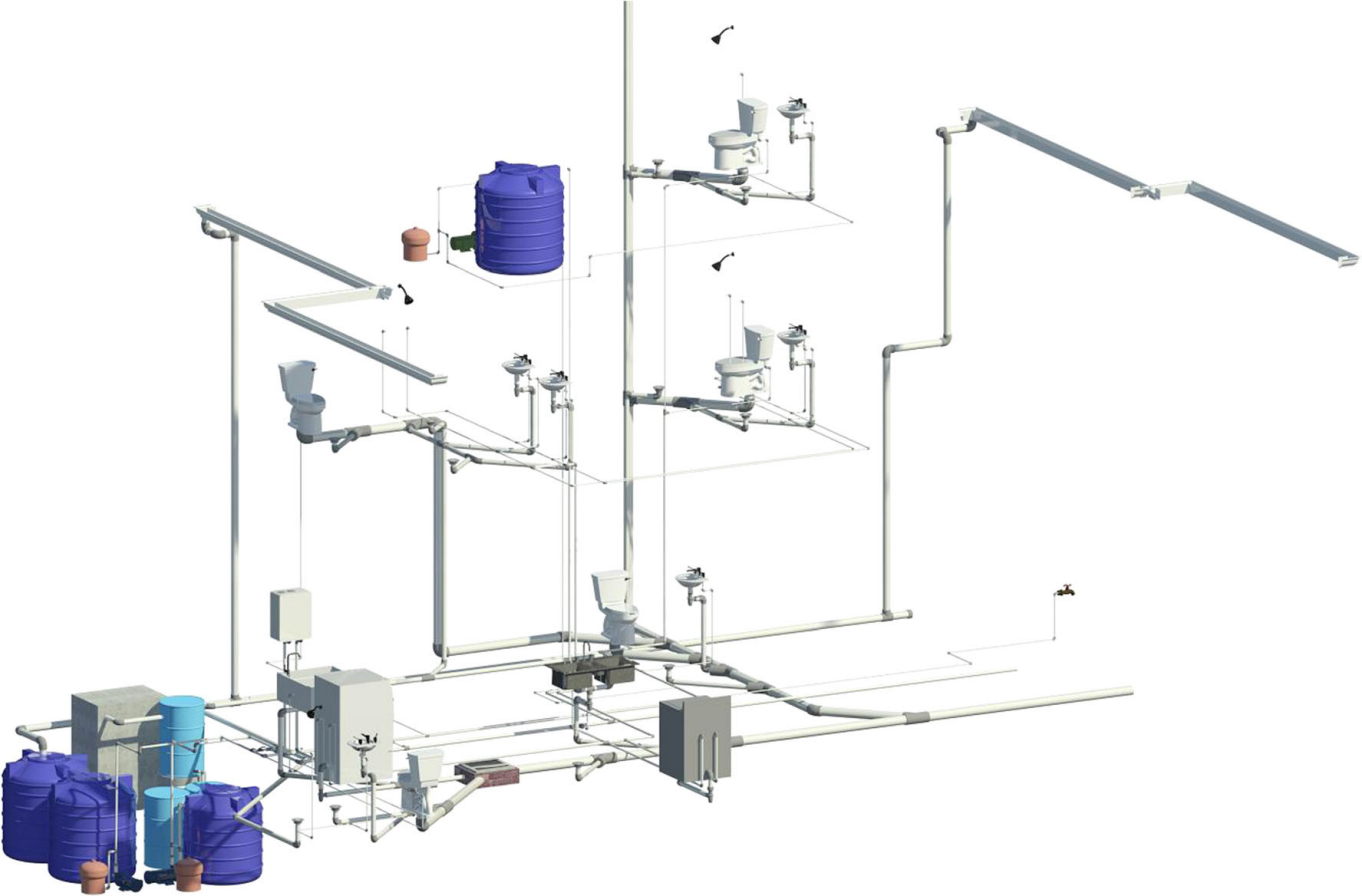
Proposed RWH and GWR system collection and distribution network design

**Fig. 6:**
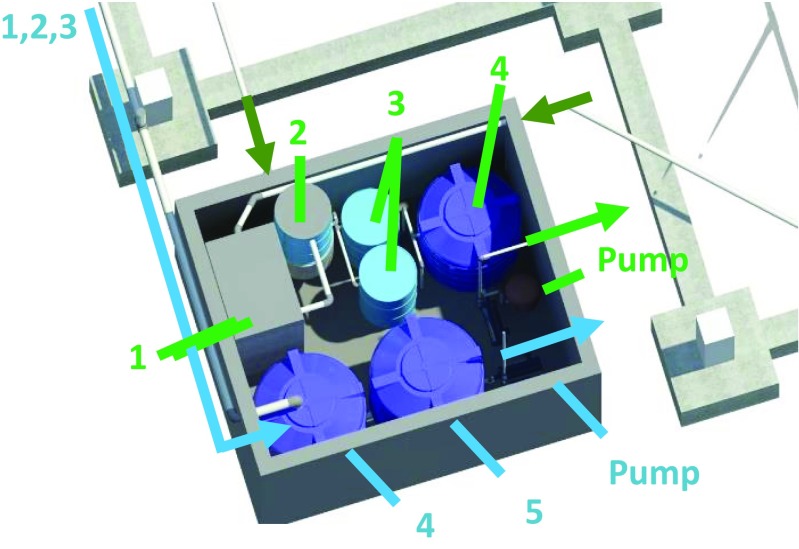
The 3D view of the RWH and GWR system machine room (*numbers* correspond to the descriptors in Fig. 7)

**Fig. 7:**
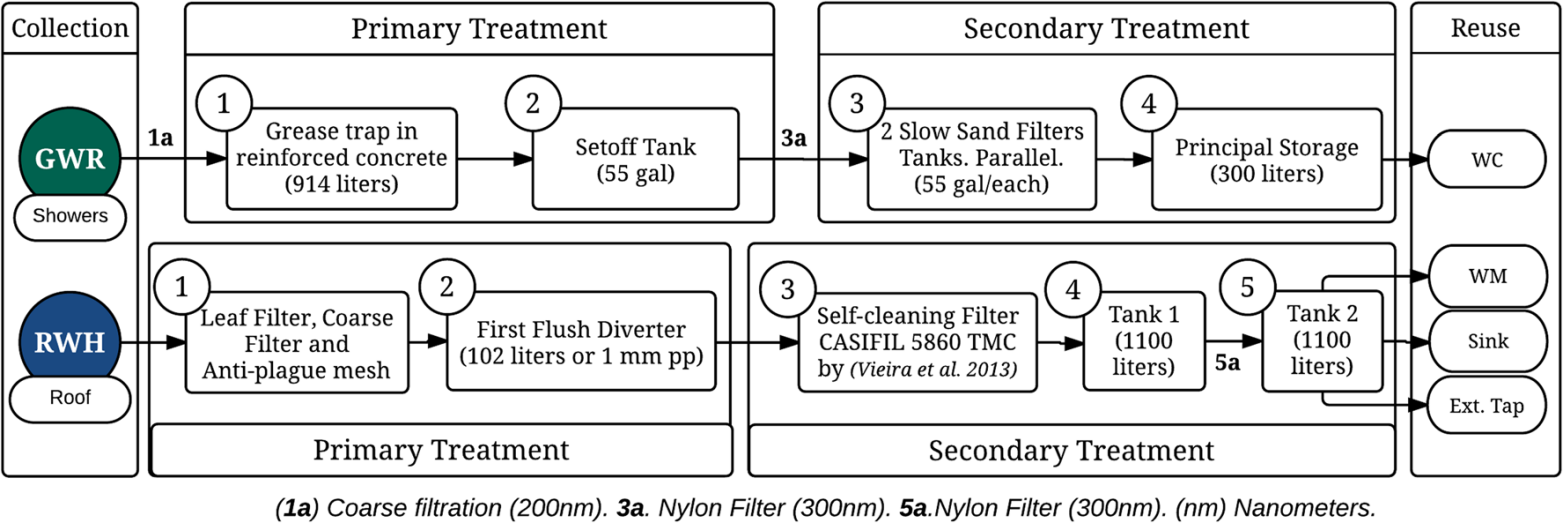
Detailed configuration of the design of the Alt 2 RWH and GWR systems

For the design of the Alt 2 system, the treatment levels considered were preliminary, primary and secondary. The design included the following technological components: grease trap, clarifier tank, slow sand filters and a storage tank. These components are the most commonly used locally and those with lower complexity for the required treatment processes. Figures 5, 6 and 7 detail the networks, the machine room and a summary of the RWH and GWR systems.

### Financial feasibility of the selected alternative

Fuller consideration of the network layout and treatment processes required by the Alt 2 RWH and GWR systems enabled a more in-depth estimation of the financial feasibility of the implementation of the system within a residential building. This brief section describes the method used to generate costs and the indicators used to assess feasibility. The following section describes the results of applying the method and indicators.

## Costs

The net construction cost was the difference between the total cost of the (conventional) original system and the Alt 2 RWH and GWR systems. The conventional and Alt 2 designs were modelled in Autodesk Revit® to estimate workloads and a budget for the total system cost. Unit prices were obtained from the ConstruData® database (a comprehensive online database of construction costs and other downloadable listings) (ConstruData 2015), as well as price lists suggested by specialised suppliers, including quotations requested by telephone.

Labour-related maintenance costs were estimated from the salary of a qualified technician (∼249 USD/month), according to the Colombian Labour Observatory and the hours needed to carry out the required tasks (i.e. between 2 and 4 h, depending on the type of maintenance).

In the case of electro-mechanical equipment depreciation, the acquisition cost was divided into its lifetime (15 years), according to the equipment providers.

The design cost of Alt 2 considered the price of a typical network design plus an increase for the alternative design (RWH and GWR), which was allocated based on the authors’ experience due to lack of local data on this particular area. All of the derived values and costs are summarised in the “Results and discussion” section.

## Feasibility

To summarise the financial results of the project and thus express its feasibility through a cash flow, the following indicators were used: payback period (PP) (a static measure of investment that allows selecting a project on the basis of how long it will take to recover the initial investment through cash flows), net present value (NPV) (the time value of money as the value of the project and the difference between the cash flow generated on the investment made) and internal rate of return (IRR) (the discount rate that produces a level of the NPV equal to zero). The values for these indicators were estimated based on income, expenses and initial investment. Revenues included savings in the payment of the drinking water and sewerage services. Expenses included equipment maintenance, depreciation and energy costs. The initial investment integrated all processes, materials and supplies needed to install the Alt 2 RWH and GWR systems.

Cash flow was projected for 50 years based on the recommendations in the regulations for life cycle assessment of environmental projects (ISO 14040 2006). Table 8 summarises the basic information for the analysis of financial feasibility.

**Table 8:** Summary of Alt 2 RWH and GWR system project information for financial feasibility

General project data	Unit	Data
Water consumption	lpcd	203
Household size	inhab	4
Roof area	m^2^	102
RWH storage	L	2000
GW storage	L	300
Concept	Increment rates	
Projected inflation in Colombia (2015)^a^	4.05%	
Drinking water cost (until year 22)^b^	6.05%	
Drinking water cost (from year 23)^b^	7.05%	
Sewer system cost^b^	4.05%	
Energy cost^b^	5.00%	
Maintenance cost^b^	4.05%	
VPN rate	4.05%	

^a^From National Department of Statistics of Colombia

^b^From water, sewerage and energy companies’ tariffs

For all factors, an average inflation of 4.05%, according to the average change over the last 10 years on the consumer price index (CPI) in Colombia, was considered.

To estimate the projected income, an effective annual rate of 6.05% was considered for the first 23 years (maximum operating point of a reservoir that supplies water during that period to metropolitan Bucaramanga). This value included an inflation of 4.05 and 2.00% surcharge to finance the current reservoir (amb 2015). After the year 23, a rate of 7.05% was considered due to a 1.00% surcharge for future infrastructure construction and 2.00% due to growth in demand that occurs to the same extent (DNP 2009).

Similarly, the revenue analysis considered that the water shortage in the country would affect energy costs due to the high dependence of the Colombian electrical system on hydro-electric plants. Therefore, a 1% increase over the baseline increase, for a total 5.05% discount rate on energy costs, was included.

## Results and discussion

Bringing together all of the Alt 2 RWH and GWR system networks, treatment and cost elements described in the previous sections enabled a cost analysis to be performed, upon which a financial feasibility assessment could then be estimated.

### Cost analysis of the Alt 2 RWH and GWR systems

Table 9 includes a comparison of the construction costs of Alt 2 against the original system, for different budget items. For example, implementing a RWH system slightly increases the rainwater collection cost due to the requirement for additional components to ensure efficient operation. However, this is offset by the slightly reduced cost associated with sewage aspects due to the reuse of the rainwater within the building.

**Table 9:** Summary of construction costs for the Alt 2 RWH and GWR systems compared to the original (conventional) system

Chapter		RWH and GWR system (USD)	Original system (USD)
1	Location and setting out on site	382.41	348.86
2	Sewage	1,160.33	1,287.64
3	Drinking water	854.52	799.30
4	Greywater collection	223.78	–
5	Greywater treatment	163.33	–
6	Rainwater collection	724.57	571.68
7	Rainwater treatment	113.16	–
8	Hydraulic and sanitary connections and devices	2,974.61	2,939.54
9	Testing and monitoring	197.36	185.63
10	Machine room	2,011.33	–
11	Hydro-sanitary and treatment design	1,960.14	653.38
	Total costs [USD]	10,765.54	6,786.03
	Additional fees (8%)^a^ [USD]	861.24	542.88
	Total investment [USD]	11,626.78	7,328.91
	System investment [USD]		4,297.87

^a^Based on authors’ judgement due to lack of data

Table 10 shows the operation and maintenance (O&M) costs for Alt 2, including the required activities (e.g. cleaning of units, maintenance, electro-mechanic and electronic elements depreciation and replacement of equipment), their unit prices, quantity per year and total annual costs (refer to the “Materials and methods” section).

**Table 10:** Annual operation and maintenance (O&M) costs for the Alt 2 RWH and GWR systems

Type	Unit price (USD)	Quantity per year	Total price (USD)
Rainwater tank cleaning (2 × 1100 L)	6.5	0.50	3.3
Greywater tank cleaning (500 L)	3.3	1.00	3.3
Greywater compensation tank cleaning (500 L)	3.3	1.00	3.3
Grease trap cleaning (filter changes included)	14.7	1.00	14.7
Slow sand filter cleaning (sand replacement included)	32.0	0.13	4.0
Gutter cleaning	3.3	1.00	3.3
Electro-mechanic and electronic element depreciation	16.0	1.00	16.0
Pumping system preventive maintenance	5.9	2.00	11.8
Electronic valve and float preventive maintenance	39.2	2.00	7.8
Total/year			67.4

Electro-mechanical equipment includes pumps for rainwater and greywater

According to the cost analysis, investment for the Alt 2 system (excluding the value of the house) was 4298 USD. This value was 63% higher compared to the original conventional system (i.e. without RWH and GWR). In this increase, the equipment located in the machine room and the design costs were the most representative factors. To put this into perspective, the increase in the cost of the Alt 2 system accounted for 1.6% of the total cost of the house (i.e. 261,352 USD for 2015). The annual maintenance cost to ensure system operation was 67 USD.

### Financial feasibility

Table 11 presents a summary of the financial projection for 50 years after Alt 2 system installation. According to the financial analysis developed, project implementation can generate a positive net cash flow from the year 23 (payback period). For the 50 years of project operation, a 6.5% IRR and NPV of 4053 USD were determined.

**Table 11:** Financial projection for the Alt 2 RWH and GWR systems in a residential building

Year	Income (USD)		Expenses (USD)		Cash flow (USD)	Net cash flow accumulated (USD)
	Drinking water	Sewerage	Energy	Maintenance		
0					−4,298	
1	99	63	3	67	92	−4208
21	341	145	9	155	322	−241
22	365	151	10	161	345	104
50	2,295	442	37	472	2,229	28,356

Most research on RWH and GWR has been developed in building types different to individual households (i.e. public facilities, office buildings, universities and residential complexes) and has mainly been undertaken for RWH or GWR separately. Other studies have identified that the RR is less when systems are implemented in multifamily dwellings compared with individual households (Friedler and Hadari 2006; Morales-Pinzón et al. 2014). However, other experiences (Cardona 2007; Sanches Fernandes et al. 2015) indicate that positive results can be achieved in individual households if the design is effective and treatment devices are available locally, which is consistent with the approach followed in this investigation.

In the studies listed in Table 12, indicators of financial feasibility for various RWH and GWR projects and the present case study are shown. The results of PP and IRR from the present investigation have intermediate values compared with the results reported by other studies (PP 11 to 44 years and IRR between 3.7 and 5.7%). The comparison includes studies in multifamily dwellings.

**Table 12:** Profitability analyses of GW and RWH reuse in diverse contexts, including the present study

Country^a^	Reference	Scale^b^	Occupancy	System	Description^c^	Results^d^
CO	Galvis (2013)	*R*	300 households, 4 inhabitants/house	GWR	*T* multiple filtration steps, flocculator	PP 11 years, IRR (15) 5.75%
CO	Estupiñán et al. (2011)	*U*	Roofs and courts	RWH	*T* filtration, decantation, activated carbon, RS 435 m^3^	PP 22 years, IRR (33) 3.7%
BR	Ghisi and Oliveira (2007)	SH	One household, three inhabitants	GWR and RWH	*T* wetland, RS 750 L, RA 202 m^2^, use to and wm	PP 29 years
SP	Morales-Pinzón et al. (2014)	GH	Two households, four inhabitants/house	RWH	*T* none, RS 3 m^3^, RA 80 m^2^, use wm	PP 44 years, IRR (50) −0.4%
SP	Domènech and Saurí (2011)	GH	One household, three inhabitants	RWH	*T* none, RS 5 m^3^, RA 80 m^2^, use to and wm	PP 37 years
EN	Ward et al. (2012)	*O*	Occupancy 300 inhabitants (actual 111)	RWH	*T* none, RS 25 m^3^, RA 1500 m^2^, use to	PP 11 years
CO	This research	SH	One house, four inhabitants	GWR and RWH	*T* slow filtration; grease trap; RS 2 m^3^; RA 102 m^2^; use to, wm, snk, et, it	PP 23 years, IRR (50) 6.50%

^a^ *CO* Colombia, *BR* Brazil, *SP* Spain, *EN* England

^b^ *SH* single house, *GH* group of houses, *R* residential building, *O* office building, *U* university

^c^ *T* treatment system, *RS* rainwater storage, *RA* roof area, *to* toilet, *wm* washing machine, *et* external tap, *it* interior tap, *snk* sink

^d^ *PP* payback period, *IRR* internal rate of return at specified year

The implementation of the Alt 2 RWH and GWR systems in this case study was financially feasible, obtaining higher return rates on the investment compared to most studies reviewed (Table 12). In addition, comparing the case study with multifamily buildings or offices, the return time was substantially lower, showing the potential of this alternative. Lee et al. (2016) indicate that one of the challenges to increase RWH in Malaysia is the implementation by governments of subsidies for initial investment. Consequently, financial indicators such as net present value and internal rate of return can demonstrate better performance. This type of incentive, implemented in contexts such as the one proposed in this study, could help to improve financial results such as the internal rate of return.

In addition, the analysis developed in this paper should be complemented by evaluating the externalities of this type of project in the social and environmental dimensions, including aspects such as those suggested by Liang and van Dijk (2010), for example, (i) social awareness towards water availability and protection, (ii) noise pollution, (iii) avoided water pollution, (iv) avoided aquifer overexploitation, (v) chemical and biological risks associated with wastewater reuse and (vi) avoided treatment water costs. Through involvement in the design process, there is potential for increasing the end-user’s ownership of and appreciation for the efficacy and financial feasibility of an installation (Ward et al. 2013), and further work is ongoing in this area. Furthermore, to increase the availability of appropriate information to facilitate decision making regarding the implementation of individual or community systems for RWH and GWR, additional studies similar to that proposed by Gurung and Sharma (2014) for RWH may be required in order to compare the financial indicators proposed in the present paper.

Complementary analysis can be developed to determine the amount of potable water displaced and runoff attenuated from entering sewers in an urban area, if a household with a roof area as described in this study captures and uses rainwater. For example, Rostad et al. (2016) show for four major US metropolitan areas (New York City, Philadelphia, Chicago and Seattle) that typical urban RWH configurations, consisting of a 100-m^2^ roof connected to a 5-m^3^ storage volume, would be able to reduce potable water demand by over 65% in all cities whilst also reducing roof runoff generation by over 75%.

The implementation of alternatives such as the one described in this study involves new approaches to civil and architectural designs for this type of household, which requires training of skilled professionals to include these new approaches. In addition, the effects of implementing such approaches on urban rainwater collection should be evaluated as a contribution to address current urban water management challenges.

## Conclusions

This article presented a financial feasibility assessment for RWH and GWR systems (‘Alt 2’) for a high water consumption household in the metropolitan area of Bucaramanga (Colombia), which utilised questionnaire, construction database, BIM data and several standard financial indicators.

Promising results were estimated for the RWH and GWR systems at the individual household scale, with additional investment costs (over the conventional system) accounting for only 1.6% of the total cost of the studied household. The estimated payback period of 23 years was lower than reported in other studies for commercial and residential buildings (individual and collective).

The RWH and GWR systems selected and analysed in this study were designed based on consultation with potential end-users, increasing the possibility of its implementation. The opinions of potential end-users were considered in aspects such as willingness to use alternative sources (rainwater and greywater), purpose for the use of alternative sources, willingness to pay for investment and O&M and willingness to carry out O&M activities.

The implementation of decentralised RWH and GWR systems and their scaling-up could have positive effects on society and the protection of water resources. These aspects should also be quantified through complementary financial feasibility and cost-benefit assessments to enable better consideration of social (e.g. health and wellbeing) and environmental aspects. Such studies could provide information for formulating public policies that enable the broader appropriate uptake of alternative water sources and supply systems in the context of developing countries such as Colombia.
